# Validation of self-reported weights and heights in the avoiding diabetes after pregnancy trial (ADAPT)

**DOI:** 10.1186/1471-2288-14-65

**Published:** 2014-05-13

**Authors:** Kathryn A Paez, Susan J Griffey, Jennifer Thompson, Matthew W Gillman

**Affiliations:** 1 Health Policy and Research, American Institutes for Research, Washington, DC, USA; 2 Social & Scientific Systems, Inc, Silver Spring, MD, USA; 3 Obesity Prevention Program, Department of Population Medicine, Harvard Medical School and Harvard Pilgrim Health Care Institute, Boston, USA

**Keywords:** RCT, Self-reported weight, Weight validation, Weight loss interventions, Overweight, Obesity, Electronic data collection

## Abstract

**Background:**

Randomized controlled trials that test the effectiveness of mobile health-based weight loss programs are attractive to participants, funders, and researchers because of the low implementation cost, minimal participant burden, and the ability to recruit participants from longer distances. Collecting weight data from geographically dispersed participants is a challenge. Relying on participant self-report is one approach to data collection, but epidemiologic studies indicate that self-reported anthropometric data may be inaccurate.

**Methods:**

We provided women enrolled in a randomized controlled trial (RCT) of postpartum weight loss after gestational diabetes with a digital scale and training to collect and report weight via a web-based survey. To validate self-reported weights and heights, we visited 30 randomly selected women in their homes, with a reference scale and stadiometer, a mean of 34 days after the self-report. We ran linear regression models to identify characteristics that were associated with underreporting or overreporting of anthropometric measures.

**Results:**

Of the 30 women we visited, 11 women (37%) were assigned to the weight loss intervention group and 19 women (63%) were in the control group. Mean age was 38.5 years (SD 4.5). The overall mean difference between participants’ self-reported weights and the weights obtained at their home visit was 0.70 kg (+1.92). Women assigned to the intervention group underreported their weight in comparison with the control group by 1.29 kg (95% CI −2.52, −0.06). The overall difference in collected to self-reported height was −0.56 cm (±1.91). No characteristics were associated with underreporting or overreporting of height.

**Conclusions:**

Our research suggests that by providing a digital scale and developing a weight collection protocol, researchers can train women to collect and record their own study weights with reasonable validity. To achieve the level of validity required for clinical trials, researchers should consider additional strategies to assure the validity of the data.

**Trial registration:**

NCT01923350.

## Background

The dramatic increase in obesity over the last two decades, affecting more than one-third of U.S. adults and 17% of U.S. children, has made this costly and serious condition a public health priority [1]. Public health strategies are needed that reach a broad audience at relatively low cost. Mobile health (mhealth) based weight loss programs are one approach to efficiently combatting overweight and obesity of individuals on a broad scale. Researchers and participants are attracted to mhealth-based interventions because of the low participant burden and cost. One of the challenges researchers face in testing web-based strategies for weight loss is collecting weight data from a group of geographically dispersed participants. Requiring participants to come into a data collection center for weigh-ins can reduce the likelihood of enrolling special populations such as working parents and other time-pressed groups, people lacking transportation, and rural residents. Home visits by researchers are one alternative to data collection, but traveling to participants’ homes is time-consuming for research staff, and home visits may make recruitment of participants from a wide geographic area impracticable.

One solution to collecting research weights in web-based weight loss studies is to rely on participant self-report. Epidemiologic studies often depend on self-reported weight and height data, but self-report of anthropometric data is seldom relied upon in clinical trials because of concerns over data quality. In epidemiologic studies, participants are asked to recall their weight from memory. The time elapsed from self-measurement to reporting is rarely, if ever, known, and weight data may be collected by interview or survey without any protocol for self-measurement. Studies comparing self-reported weight [2-9] and height [3-9] with researcher-collected anthropometric data show that individuals tend to underreport their weight and overreport their height. In the studies we reviewed, mean differences between self-reported and collected weight ranged from 1.2 kilogram (kg) to 2.9 kg, and differences in height were 0.60 centimeter (cm) to 2.2 cm. Discrepancies between self-reported and collected weight and height varied by participant body mass index (BMI), age, and gender. Women [2-9] and men [2-5] tended to underreport weight in general, but the extent of underestimation was greater in heavier women and men compared with other groups [4]. One study found that underweight women were more likely to overreport their weight compared with normal weight women [8]. Women and men tended to overestimate height [4,5,7,9], but the extent of overestimation was greater in older men and women, shorter men, and heavier women [4]. Young adult women were more likely to underreport their weight and height but no difference was found in young adult men [3].

The purpose of this study was to assess the accuracy and precision of self-reported research weights and heights in a sample of women enrolled in a randomized controlled trial, the Avoiding Diabetes After Pregnancy Trial (ADAPT).

## Methods

### Sample

The study sample was drawn from participants enrolled in ADAPT, which evaluated the effectiveness of a lifestyle intervention to reduce weight in women who had had a pregnancy with gestational diabetes mellitus (GDM) within the previous 6 months to 4.5 years. ADAPT participants were randomly assigned to receive a system of interactive electronic technology plus coaching to modify obesogenic lifestyle behaviors (the intervention group) or to a control group who received usual care. The weight loss intervention was an adaptation of the interactive obesity treatment approach (iOTA), reported elsewhere [10,11].

We recruited participants for ADAPT from the Harvard Vanguard Medical Associates (HVMA), a multispecialty group practice in eastern Massachusetts. We identified women with a recent history of GDM from the electronic medical record (EMR). Women were excluded from further screening if they were less than 18 years old, had a significant mental health disorder that could interfere with informed consent or ability to engage in the intervention, or were no longer current HVMA patients. Women identifying as non-Asian with a BMI greater than or equal to 24 kg/m^2^ or as Asian with a BMI greater than or equal to 22 kg/m^2^ were eligible for ADAPT. Six hundred and sixty-nine women with a history of GDM were identified, 393 of whom were eligible and approved for study participation by their primary care provider. One hundred and twenty seven women consented and completed the baseline survey.

To validate the accuracy and precision of the self-reported weights and heights, a research assistant made home visits to a random sample of 25 percent of the 124 women who reported a weight at the baseline data collection and at 3 or 6 months. To be eligible for a home visit, women had to live within a 50-mile radius of Boston. We planned to conduct the home visits within the two-week period after women self-reported their weight.

**Figure 1:**
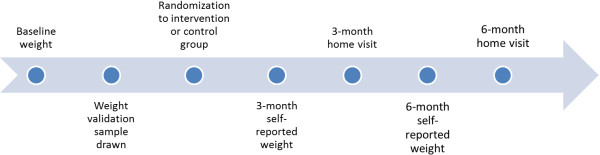
Timing of weight validation sample selection and home visits.

We randomly selected 38 women for the 3-month weight validation visit and 37 for the 6-month weight validation visit (Figure 1).

**Figure 2:**
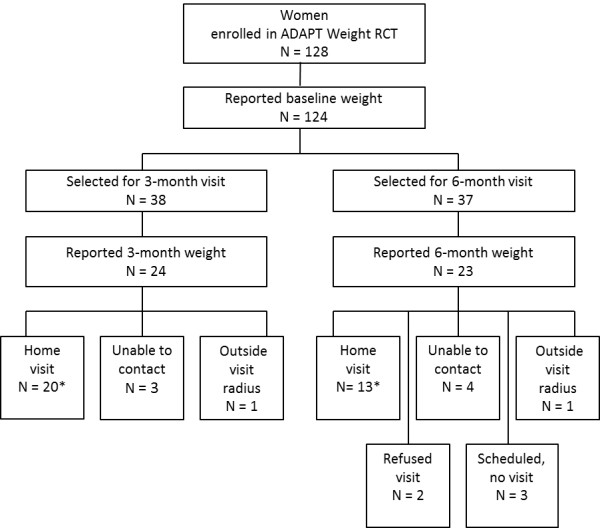
Weight validation visit eligibility flow chart.

Twenty-four of the 38 women reported their weight at 3 months, and 23 women of the 37 women reported their weight at 6 months (Figure 2). Two women were excluded from the home visits because they lived outside the 50-mile radius, and two women refused to participate. We were unable to reach seven women, and two women were scheduled but not visited. We visited 20 women following their self-report of weight at 3 months and 13 women following their self-report of weight at 6 months. We visited three women after both their 3- and 6-month weight submissions, and, from these three women, we selected one of the two visits at random for analysis.

### Procedure

As part of the ADAPT protocol, we asked women in the intervention and control groups to report their weight at 3-month intervals, beginning with entry into the study (baseline) and ending at 9 months (post-intervention). Women reported their weight by responding to an online survey question, “Please enter your weight to the nearest 10th of a pound (e.g., 165.2 pounds) from the digital scale we sent you.” Originally, we had planned to obtain height data from the EMR. When we found that height data was not consistently recorded in the EMR, we asked women to report their height, in addition to their weight, at 3 months. The baseline and 9-month (post-intervention) surveys contained additional questions about demographics, health and lifestyle, diabetes risks, and health-seeking behaviors.

We mailed women a digital scale, spare batteries, and a printed protocol with detailed weighing instructions. Trained coaches reviewed the weighing protocol with women during a phone call welcoming them to the trial. The protocol instructed women to weigh themselves in the morning before eating, drinking and dressing. For first-time use and any time after moving the scale, women were instructed to place the scale on a hard surface then calibrate it by stepping on the scale and waiting for it to blink three times. After stepping off and then back on the scale, they were to record their weight to the nearest tenth of a pound. We asked women to report weight in pounds rather than kilograms (kg) since the U.S. population is more familiar with the English system than metric system. To collect height data, we asked women to enter their height into the online survey in feet and inches or centimeters. Women were not provided with a protocol for measuring height.

The home scale used in the study was the EatSmart Precision Digital Bathroom Scale. Since our primary concern was accuracy in measuring change in weight rather than absolute weight, we tested the scale for reliability with 19 volunteers who were asked to weigh themselves at a consistent time in the morning and afternoon for 5 days. They repeated their weight twice at each data collection point and, in between, weighed themselves with and without a .91 kg weight. Weights were recorded in the following sequence: 1) weight, 2) weight with .91 kg weight, 3) weight without 2-pound weight. The mean change in weight and measurement variance for repeated measures was 0 kg (SD, 0) and for before and after weighing with 0.91 kg weight was 0.10 (+0.26); this variance was consistent with the manufacturer’s claim. The EatSmart digital scale is capable of measurements up to 181.44 kgs.

An allowable weight range of 100 to 400 pounds and a height range of 4 to 7 feet were programmed into the web-based survey instrument. During the course of the study, we reviewed the self-reported weights to identify and investigate any unusual weights that could be data entry errors. An unusual weight change was defined as a change in the first digit (hundredth place) or second digit (tenth place) from one self-report to the next. Staff attempted to contact participants directly by telephone or email to ascertain the correct weight.

After the selected women reported their 3- or 6-month weights, we contacted them by phone and e-mail to schedule a convenient time for the visit. Women were told the purpose of the visit was to check the accuracy of their home study scale and to collect height measurements. During each visit, a research assistant observed each woman’s technique for calibrating her study scale and weighing herself. Then the assistant weighed the woman using a scale of the same model to determine the accuracy of the scale issued to the participant. The research assistant measured each woman’s standing height in centimeters using a Shorr standing height board (stadiometer), following a modified version of the NHANES protocol for height measurement [12].

To analyze the data, we first ensured that scales were accurate by comparing contemporaneous home-visit measures of weight between the participant study scale and the reference scale. To assess validity of the self-reported weight report, we compared the self-reported weight and height to researcher-collected data and then compared the BMI calculated from the self-reported and researcher-collected data. We ran univariate and multiple linear regression models to identify characteristics that were associated with underreporting or overreporting weight and height using SAS version 9.3.

### Ethical approval and consent

This study was approved by the Harvard Pilgrim Health Care Human Studies Committee [Ref. no. 226175–21, ADAPT Study]; all relevant safeguards have been met in relation to patient/subject protection and are in compliance with the Declaration of Helsinki.

## Results

Women participating in the weight validation study tended to be in their late 30s, college-educated, multiparous, and married or cohabiting (Table 1). Half of the participants (n = 15) identified as white. The time lapse from women self-reporting weight to the home visit averaged 34 days (SD 20, range 12–96), with a median of 27 days.

**Table 1:** Characteristics of women participating in ADAPT who received weight validation home visits

**Characteristics**	**ADAPT sample (n = 124)**	**Weight validation sample**		
		**(ADAPT sub-sample)**		
		**Overall weight validation sample (n = 30)**	**ADAPT intervention group (n = 11)**	**ADAPT control group (n = 19)**
Age at visit, mean years (SD)	38.2 (4.2)	38.5 (4.5)	37.6 (5.2)	39.0 (4.0)
Race/ethnicity, n (%)				
White	72 (51.1%)	15 (50.0%)	7 (63.6%)	8 (42.1%)
Other	69 (48.9%)	15 (50.0%)	4 (36.4%)	11 (57.9%)
Education, n (%)				
Some college education	130 (92.2%)	26 (86.7%)	9 (81.8%)	17 (89.5%)
No college education	11 (7.8%)	4 (13.3%)	2 (18.2%)	2 (10.5%)
Married or cohabitating, n (%)				
No	31 (21.9%)	6 (10.0%)	0 (0.0%)	3 (15.8%)
Yes	110 (78.1%)	24 (90.0%)	11 (100.0%)	16 (84.2%)
Parity, n (%)				
Primiparous	30 (21.4%)	6 (20.0%)	1 (9.1%)	5 (26.3%)
Multiparous	110 (78.6%)	24 (80.0%)	10 (90.9%)	14 (73.7%)
Time from self-report to home visit, mean days (SD)	NA	34 (20)	38 (21)	31 (19)

SD = standard deviation.

NA = not applicable.

When we compared women enrolled in the ADAPT study with the weight validation sample, we found the two groups were very similar except when living arrangements were considered (Table 1). Approximately 78 percent of women enrolled in ADAPT were married or cohabiting compared with 90 percent of women who received home visits to validate their self-reported weight data. Women enrolled in ADAPT were also more likely to have taken at least some college courses compared to the weight validation sample (92.2 percent vs. 86.7 percent). Nineteen women that received home visits were from the ADAPT control group and 11 were from the intervention group. The subsample intervention and control groups differed on a number of characteristics including race/ethnicity, marital status and parity.

### Weight and height validation results

The overall mean difference between participants’ self-reported weight and the weight obtained at the home visit was 0.70 kg (+/−1.92), as shown in Table 2. When an outlier was removed from the analysis, the mean difference between self-reported and collected weight was 0.93 kg (+/− 0.27). When self-reported weight was examined by study group, we found that the control group’s mean difference in self-reported and collected weight was less than that of the intervention group by 0.6 kg to 0.7 kg.

**Table 2:** ADAPT self-reported weight/height and BMI compared with researcher-collected data (n = 30)

	**Researcher collected data**	**Self-reported data**	**Mean difference**	
			**(Collected - self-reported)**	
			**Mean of individual difference**	**Range**
	**Mean (SD)**	**Mean (SD)**		
Weight (kg)				
All	76.49 (13.6)	75.78 (13.40)	0.70 (1.92)	−5.89-3.63
			0.93 (0.27)^a^	−2.08-3.63^a^
Intervention group	75.08 (12.01)	73.99 (11.57)	1.09 (2.56)	−5.89-3.17
			1.14 (0.82)^a^	−0.82-3.17^a^
Control group	77.31 (14.67)	76.82 (14.56)	0.48 (1.47)	−2.09-3.63
Height (cm)^b^				
All	162.27 (6.59)	162.94 (6.81)	−0.56 (1.91)	−5.54-2.06
Intervention group	164.62 (7.5)	164.78 (7.75)	−0.69 (2.12)	−5.54-1.86
Control group	160.92 (5.77)	161.92 (6.22)	−0.49 (1.84)	−3.76-2.06
Body mass index (BMI), kg/m^2^				
All	29.02 (4.76)	28.35 (4.84)	0.43 (1.01)	−1.32–2.43
			0.46 (1.02)^a^	−1.32-2.43^a^
Intervention group	27.62 (3.33)	26.41 (2.52)	0.61 (0.78)	−0.75-1.61
			0.73 (0.75)^a^	−0.74-1.61^a^
Control group	29.84 (5.33)	29.42 (5.52)	0.33 (1.12)	−1.32-2.43

SD = standard deviation, kg = kilogram, cm = centimeter.

^a^Omits observation of woman whose self-reported weight at 6 months (73.5 kg) was 8.7 kg over baseline weight and 6.2 kg over 9-month weight. She had a 68-day gap from self-report to home visit data collection.

^**b**^Omits one observation where woman refused height measurement during home visit.

**Figure 3:**
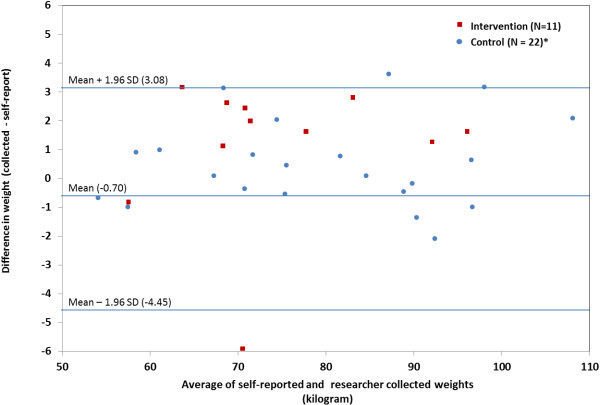
Bland Altman plot for difference in self-reported and collected weight in ADAPT intervention and control group (with 95% limits of agreement).

The extent of under- and overreporting weight compared with collected weight is highlighted by study group in a Bland Altman plot (Figure 3). The mean difference between collected and self-reported height was −0.56 cm (+/−1.91) with the intervention group overreporting height on average by 0.20 cm more than the control group, as shown in Table 2.

Body mass index (BMI) was calculated using the self-reported and researcher-collected data (Table 2). The overall difference in BMI between the collected and self-reported data was 0.43 kg/m2 (+/−1.01).

Table 3 presents the linear regression models assessing correlates of the differences between self-reported and researcher-collected weight and height. Women assigned to the intervention group underreported their weight more than the control group did by 1.31 kg (95% CI −2.41, −0.21) in the unadjusted model and by 1.29 kg (95% CI −2.52, −0.06) in the adjusted model. No characteristics were associated with underreporting or overreporting of height.

**Table 3:** Characteristics associated with differences between self-reported and researcher-collected weight and height among 30 ADAPT participants

	**Weight**^**1**^		**Height**	
	**Coefficient, 95% CI**		**Coefficient, 95% CI**	
	**Unadjusted**	**Adjusted**	**Unadjusted**	**Adjusted**
Age at visit, year	−0.08 (−0.21, 0.06)	−0.10 (−0.25, 0.05)	0.05 (−0.12, 0.21)	0.01 (−0.22, 0.23)
Race				
White	Ref.	Ref.	Ref.	Ref.
Non-white	0.57 (−0.56, 1.70)	0.22 (−1.15, 1.59)	−0.23 (−0.94, 0.48)	−0.37 (−1.66, 2.41)
Education				
Some college education	Ref.	Ref.	Ref.	Ref.
No college education	0.94 (−0.92, 2.80)	0.95 (−1.36, 3.26)	−0.88 (−2.18, 0.43)	−1.47 (−4.19, 1.25)
BMI, researcher collected	0.02 (−0.10, 0.14)	0.04 (−0.08, 0.16)	−0.03 (−0.19, 0.13)	−0.10 (−0.24, 0.13)
Time from self-report to home visit, days	−0.03 (−0.06, 0.00)	−0.01 (−0.04, 0.04)	−0.04 (−0.07, 0.00)	−0.03 (−0.09, 0.04)
Study group				
Control.	Ref.	Ref.	Ref.	Ref.
Intervention	−1.31 (−2.41, −0.21)	−1.29 (−2.52, −0.06)	−0.25 (−0.97, 0.47)	0.05 (−1.88, 1.98)
Participant survey	Ref.	Ref.	Ref.	Ref.
3 month survey				
6 month survey	−0.21 (−1.42, 1.00)	−0.50 (−1.68, 0.68)	−0.45 (−2.02, 1.11)	0.45 (−2.26, 1.35)

^1^Omits participant with unusual gain/loss.

ADAPT = Avoiding Diabetes After Pregnancy Trial.

Ref. = reference.

## Discussion

In this RCT testing an mHealth-based weight loss intervention in overweight and obese women with a history of gestational diabetes, we evaluated whether study participants could accurately collect and report weight data. Women were trained to follow a measurement protocol using a digital scale provided by the study, and they entered their weight into a web-based survey at 3-month intervals over 9 months. The mean difference between self-reported weight and weight collected by a researcher during a home visit was less than 1.0 kg. However, intervention group participants underreported weight more than control participants, by approximately 1.3 kg. This finding suggests that the intervention itself may have resulted in social desirability bias, that is, the women who received messages about weight loss felt obligated to report lower than actual weights. Thus, researchers relying on self-reported data in similar RCTs should develop additional methods to increase reporting accuracy beyond the approaches used in our study.

We also assessed the accuracy of self-reported height, but did not develop a protocol to train women how to measure height or provide a tool to do so. The average difference between self-reported and collected height was minimal and unrelated to women’s characteristics.

Unlike most epidemiologic studies examining self-reporting of anthropometric measures, our overall findings indicate that women can report weight and height with reasonable accuracy. For example, a cross-sectional study of 381 women attending a family medicine clinic found that, overall, women underestimated weight by 4.6 pounds (2.0 kg) and overestimated height by 0.1 inches [6]. Another study of 112 women recruited from general practices compared the reporting accuracy of women who were informed that their weight and height would be measured to those who were not informed and found no significant differences between the two groups [5]. Patients who expect that weight and height will be verified in a clinic setting may be more conscientious about accurately reporting their measurements than in other settings. Overall, women underreported their weight by 1.2 kg (+/−4.0), slightly higher than what we found in our study, and overreported height by 0.3 cm (+/−4.9), a level of accuracy similar to what we reported [5].

The weight validation visits were well-received by study participants. Home visits were scheduled at participants’ convenience, and each visit took fifteen minutes or less. We confirmed that nearly all participants were able to accurately demonstrate the weighing protocol. Our experience shows the importance of closely monitoring weights in real time as participants report their data. Some women in ADAPT made data entry errors of the magnitude of 10 pounds or even 100 pounds. By following up with women as data were collected, we were able to correct gross errors in a timely fashion. In future studies of self-reported weight, the data collection system would ideally question not only data entered outside of a typical weight range, but also weights reported below or above an expected range compared with earlier data entries.

Several limitations of this study should be considered. Despite our efforts to minimize the risk of data entry errors by establishing data entry parameters, some women still misreported their weight. Out of 414 data entries, we detected 12 potential errors (3%) entered by 11 women. Four of these women received home visits to validate their weight. As part of our routine quality control procedure, we were able to contact two of the four participants by phone or email and correct their weights. Of the two women who could not be reached, the researcher verified at the home visit that one had recorded her weights correctly. The fourth study participant reported a 6-month weight that was well over 6 kg higher than the baseline and 9-month weights she had entered. Therefore we excluded this woman from analysis. To examine the impact of this “outlier”, we reported the weight data with and without this woman’s weight (Table 2).

Most but not all women followed the study protocol despite our reinforcing the protocol at periodic intervals. During the home visits, we found that two women were using their personal scales, which they preferred to setting up a new scale, and one woman reported her weight in kg rather than pounds. The remaining 28 women were following the study protocol for obtaining their weight.

Underreporting weight could have been influenced by the time of day when most weights were collected by researchers. Women were asked in the protocol to weigh themselves in the morning before eating and drinking, a time period not conducive to a home visit. Most home visits took place at midday or later, when weight is likely to be higher from meals and drinks. Unfortunately we did not track the time of the home visit so could not control for time of day in the analysis. Time from report to home visit was longer than expected and could have had an influence on weight. A number of factors lengthened the average time between recording of weight and the home visit to 34 days, 20 days longer than the time period we originally designated in the study protocol. Several days passed before research staff responsible for the home visits were notified that a woman had recorded her weight. Several more days sometimes passed before women were able to be reached to schedule a home visit. Visits were also delayed or rescheduled due to participants’ vacations, travel, and personal and professional responsibilities.

In interpreting the findings it is important to acknowledge that the participants studied were overweight and obese women of childbearing age who live in a Northeastern metropolitan area. Additional studies are needed to determine if our results are generalizable to other populations.

## Conclusions

Our research suggests that, by providing women with a digital scale and a weight collection protocol, researchers can train them to collect and record their own study weights with reasonable validity. Researchers considering the use of self-reported weights for clinical trials should be prepared to frequently monitor weight data for potential data entry errors by participants and to verify any weights that seem improbable. Ideally, this review would occur automatically and at the time weight data were entered into the data collection system. Informing participants that their data may be spot-checked in home visits by research staff is one approach to improving data validity that could be tested. Another option would be to provide participants with a WiFi-enabled scale that forwards data via the internet to a research database. As WiFi technology becomes more widely available and reasonably priced, automated reporting of weights from a home scale to the research database would be a feasible option.

## Abbreviations

ADAPT: Avoiding diabetes after pregnancy trial; BMI: Body mass index; CI: Confidence interval; GDM: Gestational diabetes mellitus; Kg: Kilograms; mHealth: Mobile health; Ref: Reference; SD: Standard deviation

## Competing interests

The authors declare that they have no competing interests.

## Authors’ contributions

KAP conceived the study, participated in the design, data analysis, and interpretation of the data, and drafted the manuscript. SJG conceived the study, participated in the design and coordination, and helped draft the manuscript. JT participated in the coordination, analysis, and interpretation of data and helped draft the manuscript. MWG participated in the design, data analysis, and interpretation of the data and critically reviewed the manuscript. All authors read and approved the final manuscript.

## Pre-publication history

The pre-publication history for this paper can be accessed here:

http://www.biomedcentral.com/1471-2288/14/65/prepub
